# The validation of prediction models deserves more recognition

**DOI:** 10.1186/s12916-025-03994-3

**Published:** 2025-03-18

**Authors:** Ulrich Mansmann, Begüm Irmak Ön

**Affiliations:** Institute for Medical Information Processing, Biometry and Epidemiology (IBE), Faculty of Medicine, Pettenkofer School of Public Health, LMU Munich, Munich, Germany

**Keywords:** Prediction models, External validation, Evidence, Quality, Feasibility, Transportability, Clinical benefit

## Background

Clinical prediction models (CPM) emerge increasingly across a range of medical fields. They guide patient diagnosis, risk stratification, and optimize individual treatment or life decisions. With major algorithmic advances, an industrial-level production of CPMs is expected. But, how do we know if these models are fit for purpose and will not harm patients when applied in clinical practice?

*Validating a CPM means establishing that the model works satisfactorily for patients other than those from whose data it was derived* [1]*.* Internal validation, performed on the same patient population on which the model was developed, focuses on reproducibility and overfitting. External validation, performed on a new set of patients from a different location or at a different timepoint, focuses on transportability and benefit.

Methodology for the development, evaluation and implementation of CPMs do exist [2]. But, these concepts are not aligned into a broadly accepted good practice for CPM evaluation and implementation. There are at least three levels of evidence for the validation of a CPM: Assessing accuracy (discrimination, calibration), generalizability (reproducibility, transportability), and clinical usefulness.

In 1993, a phased process has been suggested, in analogy to those of drug development enforced by regulators before a drug is marketed [3]. Recently, Ruberg et al. propose to optimize the development of a CPM along well-established paths for diagnostic tests and physiologic biomarkers [4]. Pepe promotes confirmatory trials addressing the question whether incorporating the CPM in clinical practice improves patient outcomes [5] (Chapter 8.1.2) and recommends real-world implementations to complement such evidence. Most CPM studies in the literature investigate the feasibility of a CPM (Phase I), or develop a CPM (Phase II). But, many promising CPMs do not move to more advanced evidence phases focusing on external validation.

Despite ideas of aligning the evaluation of CPMs with that of drug development, the stringency of quality assurance for CPMs is unfortunately at a much lower level. Often, only technical validations and proof of concept studies are conducted before CPMs are established in clinical work-up and reimbursed by health insurance companies.

## Relevant issues

### What is needed for a strong validation culture?

Continuing medical education can raise awareness in the relevance and the methods to evaluate the quality of a CPM through constant training. These should be the responsibility of medical associations.

The TRIPOD AI reporting guideline for developing CPMs [6] requires not only to have clear definitions of populations, context, and methods, but also references to competing models and a justification to develop a new CPM. Researchers, should clearly communicate to which evidence a CPM investigation contributes and what it is supposed to improve. Journals and reviewers must ensure that this information is clearly visible in publications. Funders should focus on realistic validation strategies for CPM projects that are applied for.

Clinical guidelines are based on the best available evidence of diagnostic, therapeutic, and prognostic measures in clinical work-ups. CPMs will be more relevant in clinical work-ups and therefore will need conclusive evidence on their quality. Organizations responsible for development of clinical guidelines should require external validation and impact studies of CPMs. Here, systematic reviews on CPMs and meta-analyses of their external validations identify current best evidence cost-efficiently and rapidly. Independent assessments are needed whether the CPMs provide measurable benefits to patients compared to established routines. Process evaluations of CPMs need to examine their usability in joint decision-making. It is the responsibility of those who shape and regulate our healthcare system to demand comprehensive evidence on the practical aspects of using CPMs.

### What are the challenges?

In addition to the lack of awareness of the evidence on the quality of CPMs and the failure to enforce it, the complexity of the validation process poses challenges. Different levels of heterogeneity have an impact on model performance in validation: patient populations, measurement procedures, and changes in these over times. Therefore, recent literature highlights that CPMs are never truly validated and, thus, validation should be an ongoing process [7].

The current trend of personalized medicine together with rapidly evolving therapeutic options threaten CPMs to be outdated more quickly. How to validate a CPM or combine evidence in a meta-analysis when the underlying diagnostic and treatment practices are very dynamic?

For example: Using a German registry a model, a.k.a. PHREND, was developed, to predict for an individual MS patient the most effective therapy to switch to. The software is certified and implemented as a medical device. However, to our knowledge, PHREND has only limited external validation. We [8] conducted a conceptual validation and were able to show that a model following the methodological strategy of PHREND provides similar prognostic results on a large French dataset. However, it is not yet clear whether the treatment choice proposed by PHREND leads to better therapeutic results in the respective patient population. For an impact analysis, the network could set up a cluster RCT to compare patient outcomes between the decision after implementing PHREND and standard care. To show a 5% increase of success within 2 years with PHREND after switch (standard care 65%) with 80% power and 5% two-sided significance, 1380 patients are needed per group. This trial could last 4 years (2 years of recruitment, 2 years of follow-up) and appears feasible in a large network of practices. Tools to determine the optimal sample size for a robust validation study do exist [9].

### What if the current practice would continue?

Due to lack of independent validation studies, maturation of CPMs often gets stuck at an early stage. Systematic reviews of CPMs cannot be performed because respective external validation or impact studies are missing, preventing experience with CPMs and allowing misconceptions about the capabilities of such a model.

If validation studies are lacking, we need to understand its reasons and eliminate them. Without such research, the validity of CPMs’ knowledge base is in danger to be flooded by unverified performance claims. Making research decisions in favor of innovation rather than verification or consolidation leads to an accumulation of unreliable findings in the long run. The resulting disillusionment has been painfully experienced in various scientific fields [10].

## Conclusions

The scarcity of validations and impact studies hinders the emergence of critical, well-founded, organized and secure knowledge on the CPMs’ clinical value. The aim of science is not only to explore and generate new knowledge but also to verify, increase the confidence in and, if necessary, correct existing knowledge. Especially researchers with relatively small datasets should contemplate initially conducting a validation study, rather than developing a new model with insufficient sample size.

## Data Availability

No datasets were generated or analysed during the current study.

## Declarations

### Declarations

Ethical approval and consent to participate.

Does not apply.
